# The role of religious leaders in promoting acceptance of vaccination within a minority group: a qualitative study

**DOI:** 10.1186/1471-2458-13-511

**Published:** 2013-05-28

**Authors:** Wilhelmina LM Ruijs, Jeannine LA Hautvast, Said Kerrar, Koos van der Velden, Marlies EJL Hulscher

**Affiliations:** 1 Academic Collaborative Centre AMPHI, Dpt of Primary and Community Care, Radboud University Nijmegen Medical Centre, Geert Grooteplein 21, 6525 EZ Nijmegen, The Netherlands; 2 Municipal Health Service GGD Rivierenland, J.S. de Jongplein 2, 4001 WG Tiel, The Netherlands; 3 Scientific Institute for Quality of Healthcare, Radboud University Nijmegen Medical Centre, Geert Grooteplein 21, 6525EZ Nijmegen, The Netherlands

**Keywords:** Vaccination, Minority groups, Orthodox Protestants, Religion, Religious leaders

## Abstract

**Background:**

Although childhood vaccination programs have been very successful, vaccination coverage in minority groups may be considerably lower than in the general population. In order to increase vaccination coverage in such minority groups involvement of faith-based organizations and religious leaders has been advocated. We assessed the role of religious leaders in promoting acceptance or refusal of vaccination within an orthodox Protestant minority group with low vaccination coverage in The Netherlands.

**Methods:**

Semi-structured interviews were conducted with orthodox Protestant religious leaders from various denominations, who were selected via purposeful sampling. Transcripts of the interviews were thematically analyzed, and emerging concepts were assessed for consistency using the constant comparative method from grounded theory.

**Results:**

Data saturation was reached after 12 interviews. Three subgroups of religious leaders stood out: those who fully accepted vaccination and did not address the subject, those who had religious objections to vaccination but focused on a deliberate choice, and those who had religious objections to vaccination and preached against vaccination. The various approaches of the religious leaders seemed to be determined by the acceptance of vaccination in their congregation as well as by their personal point of view. All religious leaders emphasized the importance of voluntary vaccination programs and religious exemptions from vaccination requirements. In case of an epidemic of a vaccine preventable disease, they would appreciate a dialogue with the authorities. However, they were not willing to promote vaccination on behalf of authorities.

**Conclusion:**

Religious leaders’ attitudes towards vaccination vary from full acceptance to clear refusal. According to orthodox Protestant church order, local congregation members appoint their religious leaders themselves. Obviously they choose leaders whose views are compatible with the views of the congregation members. Moreover, the positions of orthodox Protestant religious leaders on vaccination will not change easily, as their objections to vaccination are rooted in religious doctrine and they owe their authority to their interpretation and application of this doctrine. Although the dialogue with religious leaders that is pursued by the Dutch government may be helpful in controlling epidemics by other means than vaccination, it is unlikely to increase vaccination coverage.

## Background

In order to effectively reach target populations, public health promotion efforts have tried to engage faith-based organizations over the past few years [1,2]. The involvement of religious leaders in health-related interventions has generally been found to improve the participation of their congregations in these interventions and thus promote positive health outcomes [3]. To reach a high level of vaccination coverage worldwide, organizations such as Unicef now advocate enhancing trust in immunization by, among other things, seeking partnership with religious leaders and groups [4]. Religious leaders are highly esteemed, and their authority can convince members of their congregations to accept or reject vaccination.

A number of epidemics started in the orthodox Protestant population of the Netherlands: a polio epidemic in 1978 (110 cases, one death) and 1992/1993 (71 cases, 3 deaths), a measles epidemic in 1999/2000 (over 3000 notified cases, 3 deaths), a rubella epidemic in 2004/2005 (almost 400 notified cases, 11 cases of congenital rubella syndrome, 2 fetal deaths) and a mumps epidemic in 2007/2008 [5-9]. All these epidemics spread to their religious counterparts in Canada [6-9]. The epidemics raised public debate about how to increase vaccination coverage among such minority groups [10-13]. In the Netherlands, the orthodox Protestant minority of 250,000 people comprises a number of denominations that separated from the Dutch Reformed Church and can therefore vary in its interpretation of the confession and its position on vaccination [14]. Some refuse vaccination because they believe it interferes with divine providence. Others accept vaccination as a gift of God. In a recent study, three clusters of orthodox Protestant denominations were identified with differing levels of vaccination coverage: high (>85%: Reformed Bond, Christian Reformed Churches), intermediate (50-75%: Restored Reformed Church and Reformed Congregations), and low (<25%: Old Reformed Congregations and Reformed Congregations in the Netherlands) [15]. The vaccination coverage per denomination was assessed at national level. At local level, however, vaccination coverage may be higher or lower than the national average for that denomination [16].

During two polio epidemics in the Netherlands in the years1978 and 1992, respectively, the Minister of Health tried to initiate a dialogue with those opposed to vaccination. In 1978, a booklet countering the religious arguments against vaccination helped fuel the discussion [17]. In some denominations, the counterarguments were clearly considered; in others, congregations were still advised against vaccination [18]. In 1992, the Minister of Health appointed a committee of three wise men who invited the principal religious leaders to discuss the vaccination issue. However, they reported that they had only had talks with representatives from two denominations, the others refused [19].

Despite such failure to communicate, the National Council for Public Health in the Netherlands advised the Minister to continue the dialogue with orthodox Protestant leaders in the expectation that once orthodox Protestant leaders are convinced of the benefits of vaccination, members of their congregations will follow [20]. Some successes of this kind have indeed been achieved in India and Africa [4,21]. But the validity of this assumption has never been checked for the situation in the Netherlands. The aim of the present study was thus to identify the role of orthodox Protestant religious leaders in promoting the acceptance or refusal of vaccination by members of their congregations. The specific research questions were as follows.

1. Do orthodox Protestant religious leaders address the topic of vaccination in their contacts with members of their congregations? If so, when and how do they address the topic?

2. To what extent are orthodox Protestant religious leaders willing to enter into a dialogue with authorities on the topic of vaccination?

## Methods

### Setting

In Protestantism, local churches are autonomous. The members of the local church choose a church council from their midst. The council consists of elders and deacons who then approach a member of the clergy to pastor their church. Following acceptance of the post and installation, the pastor conducts services, delivers sermons, organizes bible classes and confirmation classes, and provides pastoral care. The elders assist with pastoral care; the deacons manage the church finances and also help with pastoral care (e.g., financial help for members, house calls). Due to a requirement of divine vocation, the most conservative orthodox Protestant congregations in the Netherlands have very few clergy. In the local churches for these denominations today, the position of pastor is often vacant; the elders thus take over some of the tasks [22,23].

### Study design, population and procedure

Because of the explorative character of our study we chose a qualitative research design, applying the methodology of grounded theory. Semi-structured interviews were conducted with orthodox Protestant pastors who were selected via purposeful sampling. The pastors from various orthodox Protestant denominations — or the elders and deacons when no pastor was available — were approached by the researchers and invited to participate in this research. One of the researchers contacted them to explain the procedures and answer any questions. Agreement to an appointment for an interview was considered informed consent. An interviewer visited the participants in their homes to interview them with regard to numerous vaccination topics (see Table 1). The interviews lasted 60 minutes on average. The selection of new participants was driven by the analysis of the interviews, we actively sought religious leaders with different stances and practices. Moreover, interview questions were added or adapted based on the analysis of previous interviews in order to understand and test emerging concepts [24]. Inclusion and thus the interviewing of participants was continued until no new information was gleaned.

**Table 1:** Interview topics

		
**1**		Introductory questions
	a	How many people are in your congregation?
	b	How long have you had your position here?
	c	Where were you previously appointed?
	d	Do you have an idea of what the vaccination coverage in your congregation is?
**2**		Do you receive questions about vaccination from the members of your congregation?
	a	What kinds of questions?
		- Interpretation of the bible and other text
		- Personal advice with regard to decision-making
		- Doubts of conscience following illness or vaccination
	b	From whom and when?
	c	How do you handle such?
	d	More questions during epidemic outbreaks?
**3**		Do you have an idea of the decision-making process regarding vaccination in the families in your congregation?
	a	What factors are, in your opinion, decisive?
**4**		Do you, yourself, raise the topic of vaccination for discussion?
	a	During home visits?
	b	During confirmation classes?
	c	In sermons?
	d	Otherwise
	e	During epidemic outbreaks/
**5**		Do you have contact with other religious leaders on the topic of vaccination or other topics?
	a	From your own denomination?
	b	From other denominations?
	c	Regularly? Or only during epidemics outbreaks?
**6**		Have you had contact with the government about vaccination or other topics?
	a	With the mayor?
	b	With the public health service?
	c	Regularly? Or only during epidemics outbreaks?
**7**		What is your position on possibly obligatory vaccination?
**8**		Is there anything else that you think is of importance and would therefore like to add?

### Analysis

The interviews were recorded and transcribed verbatim. The transcripts were thematically analyzed using the qualitative software program Atlas.ti 6.0. Two analysts (SK and WLMR) independently coded the transcripts and subsequently reviewed, discussed, and refined the coding schemes until consensus was reached. Emerging concepts were assessed using the constant comparative method from grounded theory [24]. This means that when this concept was identified, previously analyzed interviews were reviewed in order to check if their content fitted into this concept.

## Results

Data saturation was reached after a total of 12 interviews with 7 pastors, 3 elders, and 2 deacons. Most of the interviewees belonged to denominations with nationally intermediate to low levels of vaccination coverage (see Table 2). During the inclusion phase, two other pastors were approached but refused to participate: One for practical reasons; the other considered the issue unimportant.

**Table 2:** Subgroups of religious leaders according to their role in influencing acceptance or refusal of vaccination among congregations

	**Don**’**t address topic**	**Focus on deliberate choice**	**Preach against vaccination**
N	3	4	5
Denomination	RRC	CRC	ORC
	RC	RRC	RCN
		RC	
Position	Pastor	Pastor	Elder or deacon
Estimated local vaccination coverage	High	Intermediate	Low
	(> 85%)	(50-75%)	(< 25%)
Personal decision on vaccination	Acceptance	Refusal	Refusal
Way of addressing the topic	Not applicable	Discussion	Preaching
			Teaching
Mission field	Not applicable	Pastoral care	Sermons
		Confirmation classes	Confirmation classes

*RRC* Restored Reformed Church, *RC* Reformed Congregations, *CRC* Christian Reformed Churches, *ORC* Old Reformed Congregations, *RCN* Reformed Congregations in the Netherlands.

### Influence of church order

With regard to their addressing of the subject of vaccination in contacts with congregation members, three subgroups of religious leaders stood out: those who fully accept vaccination and do not address the topic, those who have religious objections to vaccination but focus on deliberate choice, and those who have religious objections to vaccinations and preach against vaccination (see Table 2). The approach that the religious leaders apply seemed to be determined by the acceptance of vaccination in their congregation as well as by their personal point of view. This can be explained by the orthodox Protestant church order: the local congregations choose their own religious leaders, appointing pastors and elders who have views compatible with those of the members of the congregation.

### Religious leaders who do not address the topic of vaccination

The three religious leaders who did not address the topic of vaccination in their contacts with members of their congregation were pastors from either the Restored Reformed Church or the Reformed Congregations, denominations with on national level an intermediate vaccination coverage. However, they either knew that nearly all of the members of their local congregation were vaccinated or expected this to be the case. They did not receive questions about vaccination during confirmation classes or other pastoral care. Nevertheless one of these leaders sometimes raised the topic of vaccination in confirmation classes and reported that the youth did not see any religious objections with regard to vaccination anymore.

All religious leaders of this subgroup fully accepted vaccination, were vaccinated themselves, and had their children vaccinated. They considered refusing vaccination to be something of the past, the polio-epidemic of 1978 being the turning point. An older religious leader reported receiving more questions about vaccination at that time:

*Respondent 12*:

*I was always honest and let people know that we were vaccinated*. *But I can*’*t say that I served a role model function*. *I can*’*t say that the people then said* “*Oh*, *the pastor does it*, *so we should*, *too*”.

At present, in the largely vaccinated local congregations of these religious leaders vaccination is no longer an issue.

### Religious leaders who focus on deliberate choice

The four religious leaders who focus on deliberate choice all came from local congregations with a mixture of vaccinated and non-vaccinated members. In contrast to the other subgroups of religious leaders, they reported sometimes receiving questions about vaccination, particularly from young couples who did not agree on vaccination. In personal meetings they stressed the importance of deliberate decision making.

*Respondent 1*:

*Then I try to emphasize that it is a personal choice*. *You shouldn*’*t do it*, *or refrain from it*, *for me*. *It*’*s a bit of tradition but* … *you should reflect on it*. *Don*’*t just blindly follow*, *like* “*my parents didn*’*t do it*, *so I won*’*t either*.” *I really like for them to reflect and think for themselves*. *My child can later ask me*: “*Why didn*’*t you do it*?”. *I have to have an answer then*. *And I have it then*. *And others should have it then as well*.

Next to some relevant bible passages, the difficulties in the decision-making process and the psychological consequences of the decision were discussed.

*Respondent 3*:

*The rule that I*, *myself*, *follow in those sorts of situations is that you cannot place a burden on someone*’*s conscience*. *They have to find a way out*, *together*, *and respect each other*’*s standpoints*. *And things should tip to the side of the one who says* “*I can*’*t live with this*.”

These religious leaders also stimulated discussion of the arguments for and against vaccination in confirmation classes but reported rarely mentioning the topic in sermons as they considered sermons too much of a one-way affair.

Interestingly, all of these leaders reported having religious objections to vaccination, not being vaccinated themselves and not having their children vaccinated. While aiming to stimulate deliberate decision making and conscious choice they did not hide their own point of view and related their personal experiences with God rewarding their choice to not vaccinate.

*Respondent 8*:

*At the same time*, *if they ask me my personal opinion*, *I give it to them*. *We have*, *personally*, *never dared to have our children vaccinated*. *We have also always seen that the Lord takes care of us*.

Although the religious leaders of these congregations with a mixture of vaccinated and unvaccinated members personally have religious objections to vaccination, they professionally focus on deliberate choice.

### Religious leaders who preach against vaccination

The group of leaders who preach against vaccination consists of elders and deacons belonging to either the Old Reformed Congregations or the Reformed Congregations in the Netherlands, which are the two denominations with a nationally very low level of vaccination coverage. These denominations are also the denominations with very few clergy. The elders and deacons reported that vaccination is not an issue in their congregations as the doctrine is clear: Man should not interfere with divine providence.

The elders reported never receiving questions about vaccination during pastoral care.

However, one elder did mention always raising the topic himself during pre-marital consultations.

*Respondent 7*:

*During house calls we hardly ever get questions on vaccination*. *I think most people in our congregation think in the same way*. *The parents teach their children*.

*Respondent 4*:

*We talk about it during premarital consultations*. *Then people are in a stage that they have to decide on these subjects*.

In contrast to the other subgroups, this subgroup of religious leaders reported that vaccination was sometimes mentioned in sermons. The proper interpretation of the bible on the topic of vaccination is also taught during confirmation classes.

*Respondent 6*:

*Primarily during the lesson on divine providence*. *It*’*s talked about there*. *Insurance*, *vaccination*, *yeah*. *That everything is in God*’*s hands and that we should leave things up to God and that we cannot intervene*. *But*, *this is also sometimes touched upon in sermons*. *People know it*, *how things are*, *but in confirmation classes it is explained in more detail*.

### Dialogue with authorities

Regardless of the subgroup they belong to, all of the religious leaders thought that vaccination should remain voluntary in the Netherlands and also that, if vaccination is required under specific circumstances (e.g., for medical personnel), religious exemptions should be possible.

*Respondent 12*:

*No*, *I think that people should really be left free in this because there are people* — *and I also have respect for this* — *who really do not and cannot* — *on the basis of their inner convictions* — *allow it to be done*….

Although orthodox Protestants are generally law-abiding and the orthodox Protestant political party considers government to be an instrument of God [25], the respondents nevertheless emphasize that, in the case of obligatory vaccination, the laws of God overrule the laws of man and that in that case they will not obey the government.

Most of the religious leaders in our study said that they would be willing to enter into a dialogue with authorities, at least during an epidemic. They expected that a dialogue with the authorities would provide an opportunity to explain the orthodox Protestant objections to vaccination and thus add to mutual understanding. Such a dialogue, should focus on measures to control the epidemic in general. One elder, for example, reported consulting the Municipal Health Services for advice on whether to cancel a large public meeting during the 1992 polio epidemic. The respondents in our study nevertheless doubted that a dialogue specifically aimed at increasing vaccination coverage would be effective. This is because the religious leaders consider explanation of the bible and guidance with regard to the application of biblical principles during daily life to be their core business; they are therefore not willing to promote vaccination simply on the behalf of authorities.

Respondent 2

*That the government calls*, *for instance*, *for everyone to be vaccinated*….*I won*’*t let me be guided by this*. *No*, *then I think that what the government says is well*-*intended*, *but I have to look to scripture first and then to the government*. *That is what Peter*, *for example in Acts 5*, *says to the high priest* —*who represents the government in Jerusalem*. *A situation arises in which something that is not in accordance with the bible gets imposed and Peter elegantly states* “*We must obey God rather than men*.” *These moments can occur*, *thus*. *Contact is good*, *but they should not impose things on me*. *That is not in accordance with scripture*.

## Discussion

With regard to their addressing of the topic of vaccination in contacts with congregation members, three subgroups of orthodox Protestant religious leaders could be distinguished in our study: those who do not address the topic; those who focus on members of the congregation making their own deliberate choice; and those who preach against vaccination. All three subgroups nevertheless agree that vaccination in the Netherlands should remain voluntary. They are also willing to participate in a dialogue with authorities, but unwilling to promote vaccination on the behalf of authorities.

### Secularization

As far as we know, the influence of religious leaders on public health interventions in the Netherlands has not been previously studied. This is not surprising as the Netherlands is a very secularized country. In 2002 only one third of the population reported being a member of a religious congregation [26]. Religious leaders may thus not be the most appropriate intermediaries for interventions aimed at the general population because they are only in a position to reach a small portion of a country’s population. On the other hand religious leaders can help to approach minority groups with a common religion. Collaboration with Islamic religious leaders has for example been suggested to help increase living donor kidney transplantation within ethnic minority groups [27]. In a similar vein, the target population for increasing vaccination coverage is orthodox Protestant groups, which means that their religious leaders could conceivably serve as intermediaries. We found, however, that the orthodox Protestant leaders were not willing to promote vaccination.

### Church order and religious leaders’ attitudes towards vaccination

Unlike many other religions and their churches, the orthodox Protestant church order is organized in a democratic, bottom-up manner with the local congregation appointing its leaders who thus have views compatible with the majority of the members [22]. This practice is reflected in our results.

Those religious leaders who do not address vaccination all came from congregations where vaccination is no longer an issue; almost everyone , including the religious leader participating in our study , accepts vaccination. There is therefore no more need to increase vaccination coverage among the members of such congregations.

The religious leaders who preach against vaccination, in contrast, take a dogmatic stance that clearly reflects the views of most of the members of their congregation. It is very unlikely that these religious leaders will change their standpoint on vaccination and even more unlikely that such a change of standpoint would be accepted by the congregation. The church council can even dismiss a religious leader who changes position on an important issue.

Those religious leaders who focus on members making a deliberate choice are leaders who face a congregation with members in doubt. They stimulate religious argumentation and care for the psychological consequences of one’s decisions. The “open” perspective of these religious leaders is probably influenced by their specific education in pastoral and spiritual care [28]. However, these religious leaders do not only stimulate deliberate decision-making, they also provide guidance by discussing the scripture. This exegesis reflects their personal attitude towards vaccination. It is striking that all religious leaders who focus on a deliberate choice personally object to vaccination. Therefore it is expected that these religious leaders –although they stimulate a deliberate choice- do not stimulate acceptance of vaccination.

### Nature of the objections to vaccination

The successes reported by Unicef for the strategy of seeking partnerships with religious leaders are for developing countries that have just started or expanded their immunization programs and also have high levels of illiteracy [4]. The religious leaders are mainly Islamic imams and catholic priests who explain the duty of parents to secure the well-being of their children to their congregations (i.e., preach about vaccination). Another report of a successful intervention comes from the USA, where the involvement of religious leaders in the campaign to increase influenza vaccination coverage indeed increased coverage among adults [29]. This study was conducted in an underserved, inner city location that had practical barriers to attaining vaccination. Yet another successful example of partnering with religious leaders concerned the politically-motivated boycott of the polio vaccination campaign in Nigeria on grounds that the vaccine might be unsafe: Religious leaders were successfully convinced to stop the boycott once the safety of the vaccine was guaranteed by foreign biomedical experts of the same religion [21].

The situation in the Netherlands is very different than the situation in developing countries. Since 1957, all children have been offered vaccinations free-of-charge under the National Immunization Program. Socio-economic barriers are thus not relevant. National vaccination coverage is about 95% [30]. Among the orthodox Protestant population in the Netherlands, however, there has been opposition to vaccination for over 150 years and this opposition is deeply rooted in religious doctrine [10,14,22]. Unicef stresses in its manual for partnering with religious leaders the importance of seeking a case for vaccination *within* the relevant religious doctrine or holy books [4]. Among orthodox Protestants however, the topic of vaccination has been discussed over and over again, and the religious leaders all have chosen their position in this discussion, based on their interpretation of scripture. Moreover, because the interpretation and application of scripture is their core business, they owe their authority among congregation members to their religious ideas. Changing these ideas in order to help increase vaccination coverage would thus affect their credibility and undermine their authority. Although the dialogue with religious leaders pursued by the Dutch government may be helpful in controlling epidemics by other means than vaccination, it is unlikely to increase vaccination coverage.

### Possible limitations

We distinguished three subgroups of religious leaders with various attitudes and practices regarding vaccination. Because of our qualitative study design we could not assess the size of these subgroups. However, for all subgroups the role in promoting acceptance of vaccination was limited.

A possible limitation of our study is the lack of respondents from denominations with a nationally high level of vaccination coverage: Only one of our respondents represented such a group. Given that we continued to include participants until no new information could be gleaned, we do not think that inclusion of more participants from denominations with a nationally high level of vaccination coverage would alter our results. We expect the far majority of religious leaders from such denominations to fully accept vaccination, just as their congregations do, and thus fit into the first subgroup of religious leaders distinguished in our study: those who see no need to address the topic because vaccination is already accepted.

Another possible limitation is the inclusion of only elders and deacons from the Old Reformed Congregations and Reformed Congregations in the Netherlands. Their educational backgrounds are different from the educational backgrounds of the pastors included in our study. The elders and deacons also have regular jobs and thus fulfill their religious duties in their spare time. Most orthodox Protestant denominations collaborate with universities on the education of their pastors, but the few pastors in the Old Reformed Congregations and Reformed Congregations in the Netherlands are educated “on the job” by more experienced pastors, as divine vocation is the only requirement for them [22]. Given that the pastors in these denominations are also scarce and congregation members will therefore predominantly have contact with elders and deacons, we consider the elders and deacons acceptable representatives.

Finally, our data, collected by interviewing religious leaders, are per definition subjective. However, they are in line with the results of a previous study on vaccination among orthodox Protestant parents. These parents reported not consulting their religious leaders during the decision-making process [31].

## Conclusion

Orthodox Protestant religious leaders are appointed by their congregations and therefore generally hold views that are compatible with those of the majority. With regard to their role in influencing the acceptance or refusal of vaccination, three subgroups could be distinguished: those who see no need to address vaccination as it is fully accepted by their congregation; those who focus on having members of their congregation make a deliberate choice but nevertheless express their own personal objections; and those who clearly preach against vaccination. As the religious leaders owe their authority to their religious ideas and the objections they may have to vaccination are deeply rooted in religious doctrine, a major change of position on the issue could affect their credibility and undermine their authority. The dialogue with religious leaders pursued by the Dutch government is thus not likely to contribute to increased vaccination coverage. Before seeking partnerships with religious leaders for purposes of health promotion, moreover, the religious stance of the leaders with regard to a specific activity should be determined and taken into consideration.

## Competing interests

The authors declare that they have no competing interests.

## Authors’ contributions

WLMR conceived of the study, participated in the design, collected data, participated in analyses and drafted the manuscript. JLAH participated in the design of the study and helped to draft the manuscript. SK participated in analyses and revised the manuscript. KvdV participated in the design of the study and revised the manuscript. MEJL participated in the design of the study and helped to draft the manuscript. All authors read and approved the final manuscript.

## Pre-publication history

The pre-publication history for this paper can be accessed here:

http://www.biomedcentral.com/1471-2458/13/511/prepub
